# O.6.2-8 Enforcing physical education in the official examinations in Anglophone Cameroon

**DOI:** 10.1093/eurpub/ckad133.278

**Published:** 2023-09-11

**Authors:** Kemshi Suleman Mbuih, Yembe Thomas, Sama Fred Claudius, Ngondiep Tamba Rodrique, Akfua Clotilda, Wallen Forsuh, Mbuih Larissa Mana, Ngwa Maria Goretti Neng

**Affiliations:** InterCultural Alliance for Development, Cameroon; skemshi@gmail.com; InterCultural Alliance for Development, Cameroon; skemshi@gmail.com; InterCultural Alliance for Development, Cameroon; skemshi@gmail.com; InterCultural Alliance for Development, Cameroon; skemshi@gmail.com; InterCultural Alliance for Development, Cameroon; skemshi@gmail.com; InterCultural Alliance for Development, Cameroon; skemshi@gmail.com; InterCultural Alliance for Development, Cameroon; skemshi@gmail.com; InterCultural Alliance for Development, Cameroon; skemshi@gmail.com

## Abstract

**Purpose:**

Cameroon has two main educational systems: English and French, which have a colonial heritage. The controversy is that each educational sub-system has its own curriculum. In the French-speaking part of the country, sports and physical education are mandatory for obtaining the CEPE, Baccalaureate, and any other public examination. In the English-speaking part, sports and physical education are yet to be included in the curriculum. The goal of this policy paper is to change the narrative of the curriculum of public examinations in Cameroon's anglophone educational sub-system by following the example of the country's French part. This policy paper targets curriculum developers, the General Certificate of Education Board, and the Ministries of Education. What will be innovative is that students from English-speaking Cameroon will also be included in sports activities at an early age.

**Project or policy description:**

Development: We used the Health-Enhancing Physical Activity Policy Audit Tool (HEPA PAT) to collect data at the ministries of education, the Ministry of Sports and Physical Education, 197 public schools, and 204 health care facilities. Content analysis, ethnographic studies, and Strengths, Weaknesses, Opportunities, and Threats (SWOT) analysis are used to assess why average French-speaking Cameroonians have a longer life span, are physically active, and participate in more high-level sports than their English-speaking counterparts.

The Cameroon Government Certificate (GCE) of Education Board is in charge of putting this project into action. Sports and physical education should be required courses for both the ordinary and advanced levels of the GCE.

We will implement the process and conduct an impact assessment. With the process evaluation, we will monitor if the program is meeting the intended goals. The impact evaluation will give a broader view of the outcome of the program.

**Conclusions:**

When this policy is put into place, the English-speaking part of Cameroon will have HEPA and be pushed to be as healthy and fit as the French-speaking part.

